# Army Dental Surgeon and His Work in the Philippines

**Published:** 1903-06-15

**Authors:** G. D. Boak


### ARMY DENTAL SURGEON AND HIS WORK IN THE PHILIPPINES.

BY G. D. BOAK, D.D.S.

Read before the Southwestern Michigan Dental Society, April 7–8, 1903.

At the time of this writing our corps numbers about twenty in the Philippines, and we are scattered throughout the various brigades into which the division of the Philippines is divided. Upon our arrival in Manila Bay, June, 1901, after the medical inspector and custom officials convinced themselves that every thing was right, we were allowed to disembark on a launch which entered the Pasig river, and about a half a mile up we where landed at the port's wharf, and obtained our first view of Manila and the adjacent territory, which was then under military law. Manila is unlike any other city in this part of the world, so it is claimed, and belongs to a class peculiarly characteristic. Its population numbers in the neighborhood of 300,000, and is a mixture of almost every nationality. The streets are narrow and crooked, and are even worse than Boston for confusing the individual who has the sight-seeing mania. It is divided by the Pasig river into two parts, commonly called Old and New Manila, which are connected by three bridges, Bridge of Spain, a toll bridge, and a new steel structure erected as a monument of American skill and progress

Most of the business is done in New Manila, and its streets are lined with small stores, with a few exceptions, which are literally what you would call holes in the wall. Structures of wood seldom exceed two stories in height. The majority of the shops are run by Chinese, Philippinos, East Indiamen, Turks and Spaniards, with here and there an American store run in opposition to the oriental trading companies which are gradually coming under the control of Americans. Around the outskirts of the city you find the provincial style of house, the nipa shack, usually of two or three rooms, built on stilts, the building material being the male bamboo and leaves of the nipa plant or shrub tied together for shingles. Old Manila is enclosed by a high stone wall and moat built by the Spaniards about three hundred years ago; the walls are filled with secret passages which run in all directions, and I understand they put the Philippino there for safe-keeping. On the side of the walled city facing the bay runs a beautiful driveway called the "Fumeta,' which starts at Pasig river, runs up the beach to Ermita, the residence section of city since American occupancy. At the Ermita end is a small park with two large band stands; the drive is lined with tropical trees and plants making it an ideal drive in the evening, where the sweltering population pours itself out to enjoy the ocean breeze and our famous military bands. On the opposite side of walled city is another park which the Americans have beautified, and in which they have started a Zoo. Facing this park and walled city is the Government commissary for Manila, a new military ice plant, the coast artillery barracks, and the headquarters of the reserve hospital. The old-time horse-cars are still in vogue, but of instead of horses they use diminutive animals called ponies. In America we would use them to swell the out-put of a glue or phosphate factory. The principal means of transit is by a small two-wheeled concern called a carromata, pulled by a pony, at a cost of about twenty cents gold per hour. We stopped at a hotel (?) in the walled city called "The Universal," and run by a German; about the only thing universal we could find was the way they starved us at $2.50 gold per day. We reported our arrival to the Adjutant-General and Surgeon-General, and next day we received our orders and were scattered throughout the various islands, but one of our number was ordered to the reserved hospital for immediate duty, in conjunction with Dr. R. T. Oliver, our able head in Division Philippines. I was ordered to the province Pampagna, which is about forty miles from Manila on Manila

Dagupan R. R., and where the finest sugar cane is raised. I arrived just in time for the rainy season, and in my five months' stay there had the experience of going through earthquake, typhoon and flood. Rain! We in the States have never seen a real rain as compared with these, where it rains straight for a month or two at a time. I certainly thought I was going to live to see the deluge reproduced. About the time I got my office fixed up to my satisfaction in one of the above-described nipa shacks of four rooms, a three weeks' rain started in and ended in a typhoon. I had to walk to post headquarters for my meals, and while there eating supper the typhoon started; consequently, we stayed up all night and saw our kitchen blow away. Next morning bright and early I started for my hut through a small river running down the street. The sight of devastation that met my gaze quickened my steps considerably, for the better part of my house was nowhere to be seen, I afterward found all my floatable articles performing aquatic feats in the large pools of water. But with hard work and the assistance of my hospital corps man and muchacho (boy) managed to get the office in shape by 2.00 p. m. So I did all necessary work in the rain until the quartermaster could get a native to fix my roof. After a pleasant stay in Pampagna province I was "hiked" to Zambales province for three months to relieve the suffering troops stationed there. A part of this trip was made by transport, along the Zambales coast. We were landed at San Felipe in a big surf. The bank runs almost perpendicular, and we were carried ashore on the backs of natives. This province is principally covered with mountains which come down within a mile, some places two, of the sea coast, leaving a flat stretch for the industrious (?) Zambalian to cultivate. He is the nearest approach to the original inhabitant of the island I have seen. As a rule, lazy, caring only for hunting, gambling and cock-fighting, depending on his bow and arrow to feed him. After my work was about half completed, the troops where partly withdrawn from the province, and I was ordered to Tarlac province, my present station. I will mention one little incident while in San Felipe that might amuse you. The major commanding the battalion had heard that Zambales province had some man-eating crocodiles. But we were all skeptical, as we could never find the river they were supposed to occupy. We teased the president of the town a great deal about the crocodiles. About a week before leaving the president got angry and said he would get us one if he lost several men trying. Sure enough about three days after we heard a noise outside and a procession of natives came in with one measuring six feet nine inches, all bound up with a trailing vine, and presented to the senior commandant with the compliments of the president; and we had the pleasure of a crocodile hunt in the yard.

I was received with open arms, so to speak, by the soldiers. I have received nothing but courteous attention, and am on the same footing as the contract surgeon, and my shoulder-straps demand the same respect that is accorded any officer of equal rank. As one officer told me, "Your profession is a necessary evil we shall be unable to dispense with after awhile. Of course, it all depends upon our corps, however."

Being so unfortunate as to be single, I have so far been asked to mess with the post staff, consisting of the commanding officer, his adjutant, commissary, quartermaster and surgeon. Our mess costs about thirty dollars gold per month. When we are stationed within easy access of Manila, we receive fresh beef and ice from there two or three times a week. Most of our food comes from the commissary department, with the exception of such native products as we can use—beans, lettuce, shrimps, fish, eggs, chickens, vegetables; shrimps and fish are tabooed at present owing to the cholera.

Our hours of work are from 9 a. m. to 4 p. m. for officers and enlisted men. Outfits consist of five cases, besides a small library, consisting of Burchard's Pathology, American Text-book of Operative Dentistry, and Marshall's Oral Surgery. The Surgeon-General allows us three dental journals a month—*Cosmos*, *International Dental Journal* and *Dental Review*. The corps is doing a great work for the army and the advancement of the profession. Upon examination of the enlisted men in the regular army I find at least sixty percent that have come under my care have never paid any attention to their teeth. If they became bad they had them extracted. The first thing I do is to try and overcome their prejudice against dental treatment, and by so doing I not only benefit the soldier, but upon his return to civil life he will become the patient of some civil practitioner. They also come to realize the advantages gained from careful attention and cleanliness of their mouths. In the army we have a constant change from military to civil life. And if we can get not more than ten percent of this class of men educated to the necessity of proper dental treatment we shall have made another stride in the education of the masses. That the army recognized that we are doing a good work is shown by an abstract from a letter recently sent each dental surgeon through Dr. Oliver, by order of the chief surgeon. "It being a known fact that the injection of pyogenic bacteria from unhealthy mouths and teeth produce various forms of gastro-intestinal disturbances, it is recommended that dental surgeons make close investigation and report to this office any cases of diarrhœa and dysentery whose etiology points to infection from diseased oral cavities, giving the detailed account of case, hospital treatment, and an account of dental treatment received, with direct or indirect results obtained therefrom." I find my work interesting as well as instructive, as one sees many strange and exceptional cases not found in a life-time of ordinary practice. We are supplied with operating outfits only and an assistant from the army hospital corps. All men needing prosthetic work are transferred by authority of chief surgeon to the base hospitals, of which there are two: One the reserved hospital at Manila, and the other at Cebu, for Dept. South Philippines, each base station being equipped with a most complete mechanical laboratory, besides an operating office.

While on tours of duty from one place to another, we notify the commanding officer of each station one week in advance of probable date of our arrival at such station, thereby giving time for the preparation of an operating room and suitable quarters befitting our rank. Upon our arrival at stations we report to commanding officer for duty, register our names, date of arrival, number, date, source of order, and probable date of departure in a book kept at each post headquarters for that purpose, and request that all officers commanding companies or detachments at said stations send members of their command who need dental treatment to the operating room of the dental surgeon at such an hour as may be determined upon, in command of a non-commissioned officer, with a list of the names of men in squad.

Our post social duties consists of calling upon and paying official respects to the commanding officer, and afterward upon the surgeon of the post, to make arrangements for the attachments of an assistant to the hospital corps.

We are also instructed to request a list of all men at the station who were affected with syphilis, giving name, rank and company, thereby minimizing the risk of transmitting that disease to healthy patients; for, by having a previous knowledge of these cases, necessary precautions can be observed by operating upon them with instruments set apart for that purpose, and at times other than is set aside for other patients.

While the weather is by no means as hot as it is at times during the summer in the States, the average temperature for the islands during the past year being about 89 degrees F., still it is a continual heat without the invigorating change of seasons we have in the States; this gradually saps the vitality and enervates the system, producing that feeling of lassitude which is characteristic of tropical races. This enervation produces a thinning of the blood with a corresponding lessening of the resisting powers, due to the lower vitality of the individual, and especially for those who have lived previously in cold or temperate climates. That caries progresses more rapidly in this climate and the teeth more susceptible, there seems to be little doubt among the profession in the islands.

I should attribute it to the following causes, other things being equal:

1st, lowering of the vitality by a lessening of the resisting powers; 2nd, acidity of the oral secretions.

I have tested the oral secretions of about 800 patients, and of these the majority are men who had come in with the first regular regiments and had served here over two years. These tests were conducted with litmus paper, not only of the saliva as a combination of the secretions of the different glands, but I tested the secretions of the parotid, submaxillary, sublingual and racemose glands as they issued from the ducts leading into the oral cavity. With the result that in a record of 800 cases 40 percent showed marked acid reaction; 20 percent slight acid reaction; 20 percent neutral or slightly alkaline; 10 percent markedly alkaline; the reaction normally should be alkaline, which seems to indicate that this at least is a predisposing cause.

As to our disadvantages, I will speak of one instance to illustrate. The dental surgeon is ordered to a battalion of infantry for a month or two, so many soldiers, with caries complicated with necrosis of the teeth, superior molars or bicuspids missing on one side, giving only the opposite side for proper mastication, it was found upon examination that on this side we had either a six-year molar or a second bicuspid, or, in fact, both as stated above. And that if we start to treat we will not be able to get through in proper condition to fill before going to our next station as ordered. The question is, shall we extract these teeth? If so, we defeat the object of our appointment. And if we start to treat them and find that they are not in proper condition to fill, upon being ordered away you place a dressing in the roots and seal up with gutta-percha or cement, and trust to providence that no trouble will ensue. But before patient can have it attended to, being stationed at an inaccessible post, the tooth either begins to give trouble or the filling wears out, with the result that the soldier blames the dental surgeon because the tooth has never given trouble before; although we can see the minute it comes under our observation that the roots are putrescent and continually weeping pus, and if they become stopped would become abscessed. Then, as stated before, the posts are often in places inaccessible, especially in the rainy season, and a trip to a dental surgeon, even though stationed near, often entails days away from post and travel over impassible roads and rivers. So you see that it is a great disadvantage, as we found more work than we expected and the large number awaiting treatment, we had to move around more, and spend less time in one post than we do now, for the army has been decreased considerably, and we begin to understand how to handle our work better. We do not get to see the result of our work as the civil practitioner does. This is also a disadvantage which will be corrected in time by having a dental surgeon to each regiment. At present, we have little chance to correct our failures. Then, too, a man may put in a number of fillings for a soldier and send for him he finds he has been ordered away on special duty or on guard. In many instances, as we are gone from the post when he returns to his command, we do not see him again. So you see another disadvantage over here that may cause our civil brother to often criticize our work when he does not realize the condition a man is under or the circumstances surrounding the case. Many of the men are discharged with the work half completed, and, are being ordered into Manila to be sent home, left at once. Upon sending for them on day of engagement we find them on their way to the States. I only speak of these instances to let you know some reasons for conditions, you, as civil practitioners, may not understand, and how the military practice differs from civil. You must remember that in the former your patients come of their own accord, and in the latter we examine the men as a body and do the required work, as

Uncle Sam has no use for men in his service who are in any way incapacitated for duty. I think the army that has served in the islands not only realize the good that has been done, but that remains to be done in the future. During last session of Congress the bill for the reorganization of the dental corps on a commissioned basis instead of a contract corps, for the army and navy corps, has been approved by both the Surgeon General of the army as well as the navy. It shows that our work is a necessity and should be put upon a commissioned basis instead of a contract corps with dental surgeons enough, not to exceed one to every thousand men. If this or a similar bill passes, the dental profession will have scored a point second to none in its history, as we will have proved by our work and labor that the dental surgeon is a necessity for the proper treatment of the oral cavity of the soldier, on which depends to a great extent his health and physical conditions, which has obtained for him the compliments of the allied armies of China as being the finest body of troops there. Not only that, but to put the present corps of dental surgeons on a commissioned basis costs the government $1,460 less per year.

PRESENT STATUS

| | |
|---|---|
| Three examining and supervising dental surgeons, $2,520 | $ 7.560 |
| Twenty-seven contract dental surgeons, $1,800 - - - | 48.600 |
| Per year pay, total - - - - | $56.160 |
| The following commissioned basis: | |
| Three majors, $2,500 - - - - - - - - - - - - - | $ 7,500 |
| Ten captains, $2,000 - - - - - - - - - - - - - | 20,000 |
| Seventeen lieutenants - - - - - - - - - - - - - | 27,200 |
| | $54,700 |

When one realizes the amount of work accomplished and the ingenuity with which it has been done out to the minutest details, they can only say that the board has fulfilled every expectation the profession could have desired. It means no small matter to outfit thirty, or, I should say, twenty-seven men, in the short time it was accomplished besides conducting the examination of candidates for appointment, and upon reaching our last post to find every instrument as needed in its proper compartment.

